# Aberrant nuclear factor-kappa B activity in acute myeloid Leukemia: from molecular pathogenesis to therapeutic target

**DOI:** 10.18632/oncotarget.3545

**Published:** 2015-03-12

**Authors:** Jianbiao Zhou, Ying Qing Ching, Wee-Joo Chng

**Affiliations:** ^1^ Cancer Science Institute of Singapore, National University of Singapore, 14 Medical Drive, Centre for Translational Medicine, Singapore, Republic of Singapore; ^2^ Department of Medicine, Yong Loo Lin School of Medicine, National University of Singapore, Singapore, Republic of Singapore; ^3^ Department of Hematology-Oncology, National University Cancer Institute of Singapore (NCIS), The National University Health System (NUHS), Singapore, Republic of Singapore

**Keywords:** NF-κB, Acute myeloid leukemia, Leukemia, Bortezomib, Velcade

## Abstract

The overall survival of patients with acute myeloid leukemia (AML) has not been improved significantly over the last decade. Molecularly targeted agents hold promise to change the therapeutic landscape in AML. The nuclear factor kappa B (NF-κB) controls a plethora of biological process through switching on and off its long list of target genes. In AML, constitutive NF-κB has been detected in 40% of cases and its aberrant activity enable leukemia cells to evade apoptosis and stimulate proliferation. These facts suggest that NF-κB signaling pathway plays a fundamental role in the development of AML and it represents an attractive target for the intervention of AML. This review summarizes our current knowledge of NF-κB signaling transduction including canonical and non-canonical NF-κB pathways. Then we specifically highlight what factors contribute to the aberrant activation of NF-κB activity in AML, followed by an overview of 8 important clinical trials of the first FDA approved proteasome inhibitor, Bortezomib (Velcade^®^), which is a NF-κB inhibitor too, in combination with other therapeutic agents in patients with AML. Finally, this review discusses the future directions of NF-κB inhibitor in treatment of AML, especially in targeting leukemia stem cells (LSCs).

## INTRODUCTION

The nuclear factor kappa B (NF-κB) is a dimeric transcription factor which plays versatile crucial roles in a plethora of normal cellular functions by controlling a panoply of downstream genes [1-4]. This pro-inflammatory transcription factor consists of *rel* family proteins, which are related through a highly conserved DNA-binding/dimerization domain called the Rel homology (RH) domain [5]. Currently, five mammalian NF-κB family members have been identified and studied. These include NF-κB1 (p50/p105), NF-κB2 (p52/p100), RelA (p65), RelB and c-Rel [6-8]. The C-terminal regions of RelA, RelB and c-Rel contain a transactivating domain that is important for NF-κB-mediated gene transactivation, while the C-termini of p105 and p100 contain multiple copies of the ankyrin repeats, a 33-residue sequence motif, which is also found in Inhibitor of κB family members [9-11].

In unstimulated state, NF-κB complexes are sequestered in the cytoplasm by Inhibitor of kappa-B (I-κB), which mask the nuclear localization signal (NLS) of NF-κB [12-14]. Upon activation of NF-κB, an upstream IB kinase phosphorylates IκBs at the critical amino acid residues (Ser-32 and Ser-36 of IκBα; Ser-19 and Ser-23 of IκBβ), which are subsequently ubiquitinated by β-transducin repeat-containing protein (βTrCP) and then degraded by the 26S proteasome, allowing freed NF-κB dimers to translocate to the nucleus and transactivate κB-responsive elements [3, 15-17].

**Figure 1:**
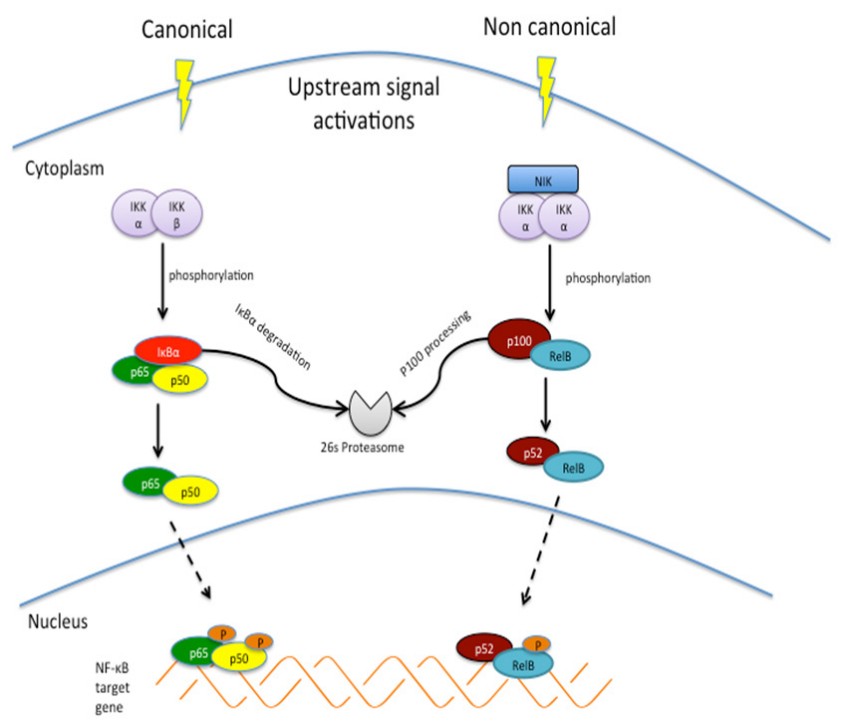
Canonical and non-canonical NF-κB signaling pathways Canonical pathway involves activation of IκB kinase [125] complex, which subsequently phosphorylates IκBα for ubiquitin mediated proteolysis, enabling phosphorylation and transient nuclear translocation of p65/p50 NF-κB heterodimer. Non canonical pathway depends on NF-κB inducing kinase [126] and IKKα complex to achieve phosphorylation–induced p100 processing, leading to RelB/p52 complex.

NF-κB signaling can occur through either the canonical or non-canonical pathways (Figure 1) [18-21]. These two pathways have different 1) activating stimuli, 2) IKK activating complexes, 3) DNA-binding heterodimers and 4) gene target [22]. The details of these two pathways were summarized in Table 1.

**Table 1:** Overview of canonical and non-canonical NF-κB pathways

NF-κB pathways	Canonical	Non-canonical
1) Activating stimuli	Tumor necrosis factor (TNF) Interleukin-1 (IL-1) Toll-like receptors (TLRs) Antigen receptors	B-cell activating factor (BAFFR) CD40 ligand Lymphotoxin βR (LTβR)
2) IKK activating complexes	IκBα, IκBβ and IκBγ complexes IκBα homodimers
3) DNA-binding heterodimers[124]	RelA:p50 (predominantly) cRel:p50	RelB:p52 (predominantly) RelA:p52 C-Rel:p52
4) Gene targets (A wide range of NF-κB target genes can be found here: (http://www.bu.edu/nf-kb/gene-resources/target-genes/)	Cytokines (TNF-α, IL-1β, IL-6, GM-CSF) Chemokines (IL8, RANTES, MIP1α, MCP-1) Adhesion molecules (VCAM-1, ICAM-1, E-selectin) Enzymes (iNOS, COX-2, PLA2)	Cytokines (BAFF/BlyS) Chemokines (BLC, SLC, SDF-1, ELC) Lymphoid organogenesis genes (PNAd, GlyCAM-1)

NF-κB signaling pathway has been shown to regulate cell survival and apoptosis. Activation of inducible nitric oxide synthase (iNOS) to increase nitric oxide (NO) has been described as a pro-apoptotic function of NF-κB activation [23-25]. However, a study by Brandão *et.al* reported high iNOS expression in blood samples of AML patients in comparison to controls, which makes this apoptotic pathway questionable [26]. It is possible that acute production of NO triggers apoptosis, in contrast, the chronic production of NO by constitutively activate NF-κB signaling could inhibit the programmed suicide [26]. On the other hand, it is generally accepted that NF-κB activation is responsible for apoptosis resistance, cell proliferation and invasiveness [27-29]. Many tumours have been reported to show upregulation of a large number of NFκB target genes, for examples, FLICE-like inhibitory protein (FLIP), Inhibitors of Apoptosis (IAPs) and some members of anti-apoptotic Bcl-2 family to inhibit apoptosis; cyclin D1, c-myc and c-myb to enhance cell proliferation; and cell adhesion molecules (ICAM-1, E-selectin), matrix metalloproteinases and several angiogenic factors such as vascular endothelial growth factor (VEGF) to promote cancer cell invasion [27, 30-35].

It has been well known that Heme oxygenase-1 (HO-1) is an evolutionarily conserved key enzyme that catabolizes free heme [36]. Free heme is lipophilic, so it causes damage in lipid bilayers of cellular membrane, intracellular organelles [37]. Thus, HO-1 has function in protecting cells from apoptosis by escalating free heme catabolism. HO-1 promoter region contains NFκB responsive element and HO-1 expression is regulated by NFκB, in collaboration with other transcription factors [38]. In AML, induction of HO-1 expression has been reported as the mechanism by which AML cells evade tumour necrosis factor-α (TNF)-induced apoptosis [39], as well as chemotherapy-induced apoptosis [40]. Therefore, it appears an attractive approach by targeting both NFκB and HO-1 for anti-AML therapy [41].

### Molecular mechanisms of aberrant activation of NF-κB in AML

Constitutive activity of NF-κB is frequently observed in different types of cancer and has been correlated with resistance of cancer cells to radiation and chemotherapies [15, 16, 23, 42-46]. Causes of such aberrant activity could be due to alterations of genes that encode NF-κB and/or its inhibitors that promote NF-κB activation; constitutive activation of IKKs that accelerate IκB phosphorylation following degradation; or exposure to inflammatory stimuli in the tumour microenvironment that constantly trigger the signaling pathway. About 40% of patients with AML have shown increased activity of NF-κB [47]. Here we will discuss various mechanisms leading to aberrant activation of this pathway in AML.

### ATM

Ataxia Telangiectasia Mutated (ATM) gene encodes a serine/threonine protein kinase, which is a master regulator of cell cycle checkpoint in response to DNA damage for the maintenance of genomic stability [48-50]. The development of AML involves multiple steps of genetic and epigenetic changes, including activation of oncogenes and inactivation of tumor suppressor genes [51]. These activated oncogenes in AML cells often induce oxidative stress (high production of reactive oxygen species, ROS) and replication stress, triggering DNA damage response (DDR) pathways, which, in turn, results in phosphorylation of ATM, CHK-1, CHK-2 and H2AX [52, 53]. In AML cells, phosphorylated (activated) ATM interacts with NFκB essential modulator (NEMO), a subunit of IκB kinase complex, and p53-induced death domain protein (PIDD) in the nucleus. Both NEMO and PIDD activate NFκB pathway [54]. Treatment of AML cells with pharmacological inhibitors of ATM or siRNA silencing ATM induces relocalization of NFκB from the nucleus to the cytoplasm, resulting in apoptosis of AML cells [54]. These results suggest constitutively active ATM leads to activate NFκB pathway in AML.

### C/EBPα

CCAAT/enhancer-binding protein alpha (C/EBPα) consists of three transactivation domains (TAD1, TAD2 and TAD3) in the amino terminus (N-termal) and a basic leucine zipper domain (bZIP) at its carboxy terminus (C-termal) for DNA binding. C/EBPα is a bZIP transcription factor, which plays a critical role in myeloid development [55-57]. The expression of C/EBPα is tightly regulated during myeloid hematopoiesis. C/EBPα expresses at low level in the HSC and terminal differentiation stage, but high at the transition stage: common myeloid progenitor (CMP) and the granulocyte-monocyte progenitor (GMP) [55, 56]. Consistent with this expression pattern, the study of C/EBPα knock-out mice shows that deletion of C/EBPα selectively blocks myeloid differentiation at transition stage and reduces formation of neutrophils and monocytes [58]. Mutations in the C/EBPα gene have been detected in 10 - 15% of patients with AML [59]. Except for some rare types of mutations, C/EBPα mutations can be classified into two main categories: (1) N-terminal mutations that lead to premature termination of protein translation, resulting in translation of a dominant negative, short C/EBPα p30 isoform; (2) C-terminal mutations that disrupt the bZIP region, resulting loss of DNA binding capacity [60, 61]. The majority of AML patients with C/EBPα mutations have double mutations, i.e., two allele carrying different types of mutations. However, some patients harbor single mutation on one allele only. Of note, only double mutations, but not single mutation of C/EBPα, are associated with favorable prognosis [59, 62]. C/EBPα and its mutant forms, harboring with N-terminal mutations or C-terminal mutations, interact with NFκB components in AML cells [63]. Several lines of evidence indicate that C/EBPα, as well as its mutant variants, interacts with NFκB p50 and induces a subset of NFκB target genes, including pro-survival Bcl-2, FLIP, through promoter binding [63, 64]. Saturating mutagenesis analysis shows that some key residues in the basic region of bZIP domain of C/EBPα is critical for the interaction with NFκB p50 [65]. The expression of C/EBPα is 3-fold lower in NFκB p50 knockout cells and p50 binds to the promoter of C/EBPα αand regulates its expression [66]. On the other hand, C/EBPα and its mutant forms can replace histone deacetylase 1 to 3 on the p50 promoter, inducing p50 expression and activating NFκB activity in AML [67].

### RUNX1

RUNX1 (runt-related transcription factor 1) is heterodimeric transcription factor belonging to RUNX gene family (RUNX1, 2, 3). RUNX1 plays a pivotal role in development of definitive hematopoiesis and primitive hematopoiesis [68-71]. Chromosomal abnormalities or point mutation involved in RUNX1 gene define a prognosis and biology distinct subset of AML patients [72, 73]. In mouse RUNX-1 deficient hematopoietic progenitor cells, both canonical and noncanonical pathways of NF-κB signaling are constitutively increased as evidenced by increased nuclear localization of p65 and p52 proteins [74]. Wild type RUNX1 binds to IKK complex in the cytoplasm and attenuates its kinase activity, thus repressing NFκB signaling. However, mutant RUNX1 forms lose the ability to inhibit IKK, leading to aberrant activation of NFκB pathway in AML cases with RUNX1 abnormalities [74].

**Table 2:** A Summary of Bortezomib in clinical trials for anti-AML therapy

Treatment regime	Subjects	Main findings	References
Bortezomib + Idarubicin +Cytarabine	9 relapsed and 22 untreated AML	Complete remission (CR): 19 cases (61%) CR with incomplete platelet recovery: 3 cases Increased CD74 expression was associated with CR. This combination regime showed a good safety profile.	Attar EC 2038
Bortezomib + Tipifamib	26 relapsed AML 1 ALL	CR with incomplete platelet recovery: 2 AML cases Both NFκB and farnesyltransferase activities were inhibited. The treatment regime was well-tolerable and had clinical activity.	Lancet JE 2011
Bortezomib + Decitabine	19 poor-risk AML patients	5 CR and 1 incompleted CR were achieved in 10 untreated cases. FLT3 was decreased via induction of miR-29 by Bortezomib. Bortezomib plus Decitabine was safe and feasible.	Blum W 2012
Bortezomib + Daunorubicin + Cytarabine	95 untreated AML	CR: 62 cases (65%) CR with incomplete platelet recovery: 4 cases (4%) This regime was tolerable and resulted in encouraging CR rate.	Attar EC 2013
Bortezomib + 17-AAG	11 relapsed or refractory AML	The combination resulted in toxicity without measurable response. Next-generation of HSP90 inhibitior might be evulated again.	Walker AR 2013
Bortezomib + Idarubicin	13 elderly patients (≥60 yrs) with newly diagnoesed AML 7 relapsed AML	CR: 4 cases (20%) Hematologic response: most of patients Treatment related-death: 1 case THis combination was safe and well tolerated.	Howard DS 2013
Bortezomib + Lenalidomide	9 AML patients 14 MDS patients	The treatment regime was tolerable. Some reponse was observed.	Attar EC 2013
Bortezomib + Azacitidine	23 relapsed or refractory AML	Complete remission (CR): 5 cases (22%) CR with incomplete platelet recovery: 3 cases The combination was tolerable and active.	Walker AR 2014

MDS: myelodysplastic syndromes; ALL: acute lymphoblastic leukemia

### TNF-α

Tumor necrosis factor-alpha (TNF-α) is a type II transmembrane protein and the soluble form of TNF-α is secreted by immune systems including macrophages, monocytes, neutrophils, T-cells, nature killer (NK)-cells in response to inflammatory stimulation [75, 76]. TNF binds two TNF receptors (TNFR1 and 2) and activates the canonical NFκB pathway [16]. In an AML mouse model, leukemia-initiating cells (LICs) or leukemia stem cells (LSCs) exhibit autocrine TNF-α secretion, which causes constitutive activation of NFκB activity in this unique cell population [77]. This finding is further supported by the positive correlation between NF-κB activity and autocrine TNF-α in human AML samples [77].

### Oncogenic kinase activation

RAS protein family includes H-RAS, N-RAS and K-RAS, which are small GTPase proteins [78]. RAS proteins transmit signals from extracellular growth factors by cycling between inactive GDP-bound and active GTP-bound states. N-RAS or K-RAS mutations occur in approximate 20% of AML cases [79, 80]. However, aberrant RAS signaling has been detected in 40% of cases in addition to RAS mutation, primarily due to is somatic mutations in the other receptor tyrosine kinase like FLT3 and c-Kit [79, 80].

Birkenkamp and co-workers observed a significant association between constitutive NFκB DNA-binding activity and persistent RAS signaling in AML blasts [81]. In *ex vivo* experiments, AML blasts with high NFκB DNA-binding activity underwent less or no spontaneous apoptosis, compared to AML cases with no or low nuclear NFκB expression. By using small molecular inhibitor Ly294002 targeting PI3K/AKT pathway, L-744832 targeting RAS, PD98059 targeting ERK/MAPK signaling and AG1296 targeting FLT3, the authors found that NF-κB DNA-binding activity was inhibited only by RAS and PI3K/AKT inhibitors, thus concluded that increased NF-κB activity was regulated by RAS signaling, but not ERK and FLT3 pathways [81]. In contrast, several other studies clearly demonstrated that either FLT3 overexpression or FLT3 mutation increased NFκB activity in AML [82, 83]. Takahashi and colleagues showed that overexpression of FLT3 in BaF3 cells activated NFB reporter and increase level of IL-6, a NFκB target gene [82]. They also showed a modest positive correlation between FLT3 and IL-6 mRNA expression in AML samples [82]. Similarly, Grosjean-Raillard *et al*. reported that constitutive activation of FLT3 signaling resulted in activation of NF-κB, while inhibition of FLT3 signaling either by small molecule inhibitor or knockdown of Flt3 with RNAi reduced NF-κB activity and induced apoptosis in AML cell lines and CD34+ primary AML cells [83]. Furthermore, comprehensive biochemical experiments revealed the underlying mechanism of NF-κB activation in which FLT3 kinase physically bound and phosphorylates IKK2, an upstream regulator of canonical NF-κB pathway [83]. In addition, Internal tandem duplications of FLT3 (FLT3-ITD), one of the most common genetic abnormalities in AML [84, 85], induced expression of RelB and p52/NF-B heterodimers, thus promoting non-canonical NF-κB pathway [86]. In summary, activation of both canonical and non-canonical NF-κB pathways appears to be an important event contributing to the leukemic transformation initiated by some crucial oncogenic kinases.

### NF-κB as a target for anti-AML therapy

Because a large body of evidence supports the important role of NFκB as a “hallmark of cancer”, there has been tremendous focus on the development of NFκB inhibitors for cancer treatment in both the academic community and the pharmaceutical industry [87-89]. Different NFκB inhibitors have been classified into 8 groups according to their chemical nature and have been reviewed in details [90-92]. Here, we focus on the proteasome inhibitor, Bortezomib (Velcade^®^, Millennium Pharmaceuticals) and other promising NFκB inhibitors in clinical trials for treatment of AML.

Bortezomib is the first-in-class proteasome inhibitor, which has been approved by FDA (USA) to treat multiple myeloma and now relapsed mantle cell lymphoma too [93-95]. Although the models of action by proteasome inhibition are not fully elucidated, one of the important mechanisms associated with the anti-myeloma functions of Bortezomib is its ability to suppress the NF-κB signaling pathway [96]. IκB, a cellular inhibitory protein of NFκB, is targeted by ubiquitin-proteasome pathway for degradation when it is phosphorylated at serine residue 32 and 36. Inhibition of the proteasome pathway by Bortezomib has been shown to block the degradation of IκBα, thus sequestering NFκB in the cytoplasm and preventing NFκB nuclear translocation and activation of NFκB target genes [97-99]. Because of its ability to inhibit NFκB activity, it provides a rationale to examine the effectiveness of Bortezomib either used alone or in combination with other drugs against AML in various clinical trials. Early phase I trial in AML evaluated Bortezomib as monotherapy in refractory or relapsed acute leukemias. As a monotherapy, the maximum tolerated dose (MTD) of Bortezomib was 1.25 mg/m^2^, and was shown to have transient hematological improvements in some patients [100]. In the subsequent trials in AML, Bortezomib was further investigated in combination with other agents. Eight clinical trials that enrolled majority of patients with AML aimed to evaluate the benefit of combination of Bortezomib with other drugs (Table 1) [101-108]. Two trials that combined the use of Bortezomib with Cytarabine and Anthracyclines (Idarubicin or Doxorubicin) showed a complete remission (CR) rate of 61% and 65%, respectively, and a good safety profile [101, 102]. One study of Bortezomib with Idarubicin in high risk of AML patients achieved a CR rate of 20% [106]. The other five trials that co-administrated Bortezomib with other targeted drugs or epigenetic drugs did not produced encouraging CR rate. But, one important note should be taken into consideration is that the subjects in these trials were high-risk patients with either refractory or relapsed AML or older than 60 years. These may be confounding factors that adversely impact on the clinical benefits of these combination therapies. In general, if these combination regimes are well tolerated, they should be further evaluated in standard risk patients. When new generation of inhibitors are developed, they might be tested in the combination regimes too.

## CONCLUSIONS

Over the last decade, our understanding of NFκB signaling and our ability to target it has evolved significantly. Although there are now 8 different classes of more than 700 NFκB inhibitors, only a few of them have advanced into clinical trials for treatment of AML. At the same time, a growing body of evidence suggests the existence of leukemia stem cells (LSCs) in AML leading to the potential relapse of disease and treatment failure [109-111]. LSCs reside mostly in a quiescent cell cycle state, which is similar to their counterparts, the normal hematopoietic stem cells [112-114], thus escaping from the effects of standard chemotherapy drugs which usually target proliferative cells. NF-κB activity is aberrantly increased in primitive human leukemia cells compared to normal primitive bone marrow cells [47, 77, 115, 116], thus it provides a novel concept to treat AML by targeting the difference between HSCs and LSCs as exemplified by the different NF-κB activity between them. [117-120]. In fact, the small molecule NF-κB inhibitor dimethylaminoparthenolide (DMAPT /LC1) has been shown to selectively eradicate AML LSCs in the laboratory [92, 121-123] and is currently evaluated in a phase I-II ‘first in man’ clinical study at Cardiff University, UK (http://medicine.cf.ac.uk/person/dr-steven-knapper/research/). Hopefully, the result of this much anticipated trial will demonstrate effectiveness of NF-κB inhibitor in AML patients.
